# Patient and economic impact of implementing a paediatric sepsis pathway in emergency departments in Queensland, Australia

**DOI:** 10.1038/s41598-022-14226-6

**Published:** 2022-06-16

**Authors:** Robin Blythe, Paula Lister, Robert Seaton, Amanda Harley, Luregn J. Schlapbach, Steven McPhail, Bala Venkatesh, Adam Irwin, Sainath Raman, the Queensland State-wide Sepsis Collaborative, Luregn Schlapbach, Amanda Harley, Adam Irwin, Nicolette Graham, Fiona Thomson, Kieran Owen, Kirsten Garrish, Emma Sampson, Debbie Long, Shane George, Keith Grimwood, Christa Bell, Bethany Semple, Claire Adams, Josea Brown, Louise Maloney, Paula Lister, Scott Schofield, Clare Thomas, Liam Dejong, Esther Bently, Lambros Halkidis, Cheryl Bird, Matthew Smith, Pia Alexander, Laura Davidson-West, Titiosibina Ebenezer Adegbija, Alice Brandt, Bree Walker, Andrea McLucas, Adam Philip Michael, Samantha Hoole, Candice Bauer, John Sutherland, Douglas Gordon Thomas, David Van der Walt, Jessica Hulme, Kerrie Burke, Helena Cooney, Doug Morel, Louise O’Riordan, Samantha Fairless, Megan Bool, Nandini Choudary, Shalini Arora, Ben Lawton, Jo Farrell, Penelope Prasad, Rudesh Prasad, Laura O’Connor, Timothy Butters, Peter Kennedy, Hanh Pham, Maya Aoude, Sara Blundell, Natasha Willmett, Louise McGrath, Karen Smith, Kate Weller, Trina Maturanec, Michael Rice, Balasubramanian Venkatesh, Paul Lane, Robert Seaton, Donna Mason, Naitik Mehta, Vikrant Kalke, Damien Jones, Mathew Ames, Mary Steele, Amy Wilkinson, Kristen Gibbons, Patricia Gilholm, Endrias Ergetu, Rachel Treadwell, Tahlia Van Raders, Jessicah Minogue

**Affiliations:** 1 Australian Centre for Health Services Innovation, Centre for Healthcare Transformation, School of Public Health and Social Work, Faculty of Health, Queensland University of Technology, 60 Musk Ave, Kelvin Grove, Brisbane, QLD 4059 Australia; 2 Queensland Paediatric Sepsis Program, Children’s Health and Youth Network, Children’s Health Queensland, Brisbane, Australia; 3 Paediatric Intensive Care Unit, Sunshine Coast University Hospital, Birtinya, Australia; 4 Department of Health, Clinical Excellence Queensland, Brisbane, QLD Australia; 5 Child Health Research Centre, The University of Queensland, Brisbane, QLD Australia; 6 Critical Care Nursing Management Team, Queensland Children’s Hospital, Brisbane, QLD Australia; 7 School of Nursing, Midwifery and Social Work, University of Queensland, Brisbane, QLD Australia; 8 Department of Intensive Care and Neonatology, and Children’s Research Centre, University Children’s Hospital Zurich, Zurich, Switzerland; 9 Digital Health and Informatics, Metro South Health, Brisbane, QLD Australia; 10 The George Institute for Global Health, Sydney, NSW Australia; 11 Department of Intensive Care Medicine, Wesley Hospital, Brisbane, QLD Australia; 12 The University of Queensland, Brisbane, QLD Australia; 13 The University of Queensland Centre for Clinical Research, The University of Queensland, Brisbane, QLD Australia; 14 Infection Management and Prevention Service, Queensland Children’s Hospital, Brisbane, QLD Australia; 15 Paediatric Intensive Care Unit, Queensland Children’s Hospital, South Brisbane, QLD Australia; 16 Queensland Children’s Hospital, Brisbane, Australia; 17 Gold Coast University and Robina Hospital, Robina, Australia; 18 Sunshine Coast Universtiy Hospital/Nambour Hospital, Birtinya, Australia; 19 Cairns Hospital, Cairns, Australia; 20 Rockhampton Hospital, Rockhampton, Australia; 21 Bundaberg Hospital, Bundaberg, Australia; 22 Redland Hospital, Cleveland, Australia; 23 Redcliffe Hospital, Oxford, UK; 24 Ipswich Hospital, Brisbane, Australia; 25 Logan Hospital, Logan City, Australia; 26 Hervey Bay Hospital, Pialba, Australia; 27 The Prince Charles Hospital, Chermside, Australia; 28 Caboolture Hospital, Caboolture, Australia; 29 Mackay Hospital, Mackay, Australia; 30 Clinical Excellence Queensland, Brisbane, Australia; 31 Faculty of Medicine, Child Health Research Centre, The University of Queensland, Brisbane, Australia

**Keywords:** Health care economics, Health policy, Paediatrics, Paediatric research

## Abstract

We examined systems-level costs before and after the implementation of an emergency department paediatric sepsis screening, recognition and treatment pathway. Aggregated hospital admissions for all children aged < 18y with a diagnosis code of sepsis upon admission in Queensland, Australia were compared for 16 participating and 32 non-participating hospitals before and after pathway implementation. Monte Carlo simulation was used to generate uncertainty intervals. Policy impacts were estimated using difference-in-difference analysis comparing observed and expected results. We compared 1055 patient episodes before (77.6% in-pathway) and 1504 after (80.5% in-pathway) implementation. Reductions were likely for non-intensive length of stay (− 20.8 h [− 36.1, − 8.0]) but not intensive care (–9.4 h [− 24.4, 5.0]). Non-pathway utilisation was likely unchanged for interhospital transfers (+ 3.2% [− 5.0%, 11.4%]), non-intensive (− 4.5 h [− 19.0, 9.8]) and intensive (+ 7.7 h, [− 20.9, 37.7]) care length of stay. After difference-in-difference adjustment, estimated savings were 596 [277, 942] non-intensive and 172 [148, 222] intensive care days. The program was cost-saving in 63.4% of simulations, with a mean value of $97,019 [− $857,273, $1,654,925] over 24 months. A paediatric sepsis pathway in Queensland emergency departments was associated with potential reductions in hospital utilisation and costs.

## Introduction

Sepsis is a life-threatening condition defined as infection that results in organ dysfunction due to a dysregulated host response^1^. In 2017, an estimated 25 million cases of sepsis were noted globally in children, and over 3 million died^2^. In Australia and New Zealand, up to 6.5 cases per 100,000 of the paediatric population will develop severe sepsis requiring intensive care unit (ICU) admission^3^. The direct costs related to children with sepsis requiring ICU admission in Australia and New Zealand was estimated in 2013 at AUD$30.7 m annually^4^. Even when children survive sepsis, the longer-term impact of sepsis and post-discharge complications can be severe^5,6^. Timely recognition and initiation of appropriate interventions in children with sepsis has been shown to save lives and improve quality of life^7^.

Implementation of sepsis protocols and institutional pathways represent best practice recommended by the Surviving Sepsis Campaign, but require substantial investment^8^. Protocolized sepsis care is rarely cost-saving^9^. In adults, economic evaluations of the Surviving Sepsis Campaign^10^, Multiple Urgent Sepsis Therapies protocol^11^, and mandated protocolized sepsis care^12^ reported improved mortality, but increased costs^13^. Paediatric sepsis quality improvement studies have focused on outcomes including survival and length of stay (LOS)^14^ but have not assessed cost impacts and were often single-centre. It is urgent to assess the cost impact on the health system of paediatric protocolized sepsis care.

We conducted a population-level economic analysis of paediatric patients treated in the Queensland Sepsis Breakthrough Collaborative. This was a quality improvement project initiated by the Queensland Department of Health in response to the Australian National Action Plan for Sepsis^15,16^. The sepsis collaborative implemented evidence-based pathways in Emergency Departments (EDs) in Queensland for earlier recognition and treatment of sepsis in adults and children aimed at improving sepsis outcomes^17,18^. The primary objective of this study was to investigate the economic impact on the wider health system of Paediatric Sepsis Pathway (PSP) implementation. In the absence of patient-level data, aggregate data was used to examine sepsis-associated hospitalizations in Queensland, Australia.

## Methods

### Study design

This study was a population-based multi-site, data-driven simulation model and cost–benefit analysis of acute service utilisation before and after the implementation of an ED PSP. The study was approved by research ethics and governance, including a waiver of individual consent (Children’s Health Queensland HREC/18/QRCH/167) as well as state Public Health Act approval. No identifying information was used, and all research was conducted in accordance with relevant guidelines and regulations. Details of the Queensland Sepsis Collaborative^18^ and the PSP^17^ have been previously described. The Consolidated Health Economic Evaluation Reporting Standards (CHEERS) checklist^19^ was used to report outcomes (Supplement 2).

### Intervention

The PSP was introduced as a paper-based clinical decision support tool, based on the Surviving Sepsis Campaign and comprised of: systematic screening of paediatric patients to facilitate recognition of possible sepsis; appropriate escalation using decision trees; a protocolized treatment bundle with guides for antibiotic prescription and administration; and a parental information leaflet^17,18^. The purpose of the PSP was to assist ED physicians to recognize sepsis earlier in the disease process, enabling earlier treatment and better compliance with the sepsis treatment bundle, leading to improved clinical outcomes. Emergency physicians were trained to use the PSP by a dedicated paediatric sepsis clinical nurse consultant. A detailed description of the PSP version 1, used during the study period, can be obtained by contacting the corresponding author, although this has since been superseded by updated versions which can be obtained at: https://clinicalexcellence.qld.gov.au/priority-areas/safety-and-quality/sepsis/sepsis-resources/paediatric-sepsis-pathways.

Study participation was voluntary and restricted to EDs capable of advanced resuscitation, stabilisation and rapid transfer^20^. Further description of the Queensland hospital capability framework is provided in Supplement 3. Twenty-one Queensland hospitals were initially eligible for involvement, and 16 chose to participate. Participating sites had nominated sepsis teams responsible for leading implementation, supported by central quality improvement advisors from the sepsis collaborative.

### Setting and population

Queensland is the second largest state in Australia geographically and has an estimated population of 5.2 million (June 2020). It has the greatest population dispersion of any Australian state, over a geographical area larger than Alaska. The proportion of children (0–14 years) in the state has been relatively steady over the last 5 years at 19.3%, reflecting a paediatric population of approximately one million^21^. Paediatric care in Queensland is centralized, with quaternary care occurring in a single children’s hospital in Brisbane. Children requiring escalation of care are transported to more specialized centres, with the mode of travel (road, rotary or fixed wing transport) dictated by transport distance.

Inpatient episodes for all patients aged under 18, admitted via ED with an ICD-10 coded diagnosis of sepsis across Queensland, were compared before and after the intervention. Patients with ward-acquired sepsis codes were excluded. Sepsis coding is provided in Supplement 3. Three hospitals piloted the PSP before it was introduced at a further 13 sites, including the state children’s hospital. These 16 sites were compared to 32 hospitals that did not implement the PSP. Sites were categorized into:

PSP-CH: The only dedicated children’s hospital (CH) in the state; implemented the PSP in the ED. This is the paediatric tertiary/quaternary hospital in Queensland and is the primary transfer destination for paediatric patients requiring a significant escalation in care.

PSP-mixed: Two dedicated paediatric EDs and 13 EDs treating a mixed population of adults and children that implemented the PSP. Does not include PSP-CH.

Non-PSP: EDs of 32 acute care hospitals that did not implement the PSP. These include 24 smaller regional facilities without ICU capability and eight tertiary facilities with ICU capability.

**Figure 1:**
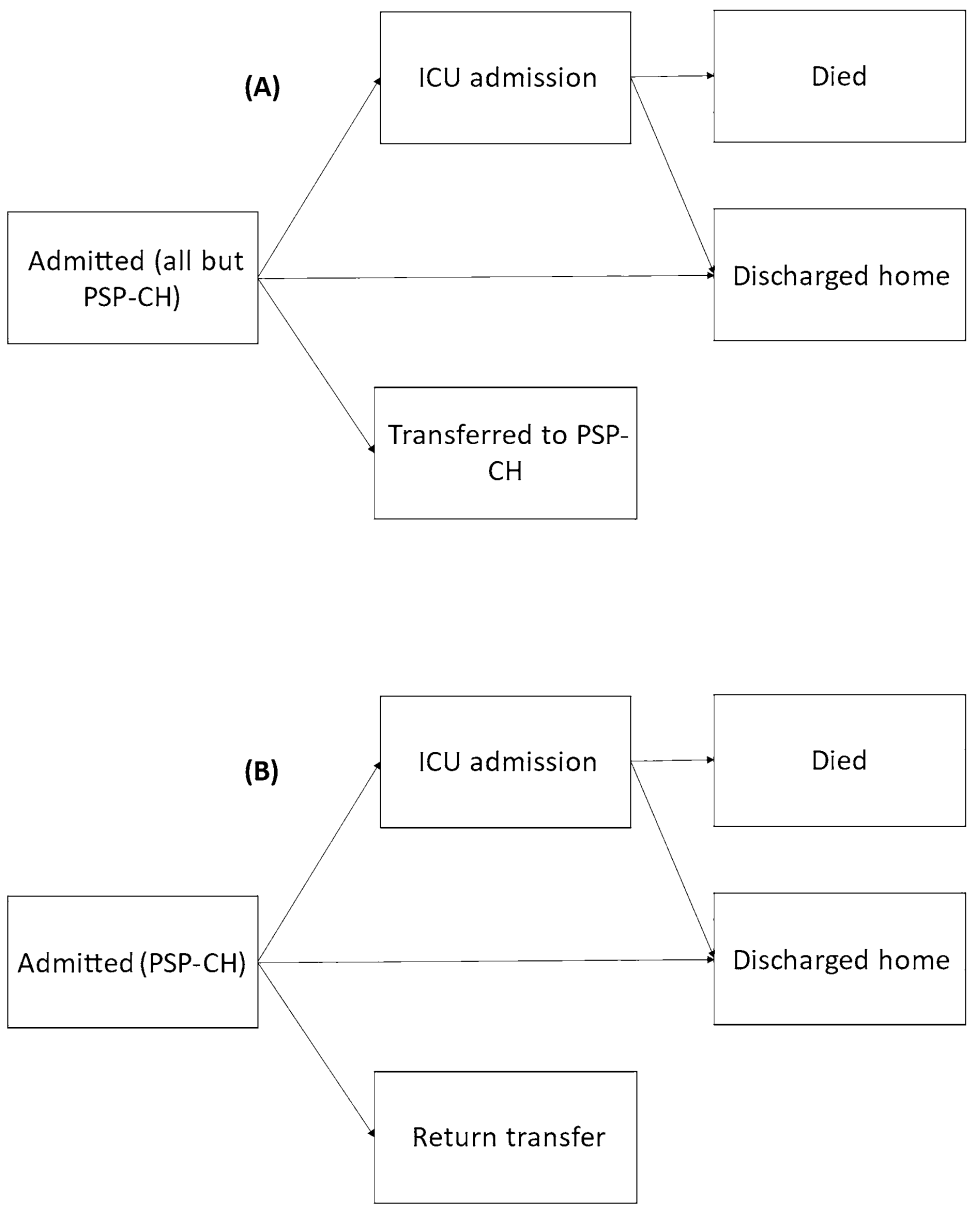
Admission pathway for all hospitals except PSP-CH (**A**) and PSP-CH only (**B**). PSP-CH is the only specialist children’s participating hospital.

Figure 1 displays the likely patient pathways for PSP-mixed and non-PSP sites (panel A) and the PSP-CH (panel B). Patients were assumed to be non-palliative, meaning that mortality was only likely in ICU. In Queensland, the transfer process typically occurs by local referral from lower capability centres to an intensive care specialist through Retrieval Services Queensland. The intensivist provides advice, initiates triage with logistic support by Retrieval Services Queensland, and facilitates transfer to a higher capability tertiary care centre if required. This transfer is then conducted by paediatric intensive care, a designated adult, or paramedic team depending on clinical need.

### Study periods

The pre-implementation period was from September 2015 to August 2017 for one PSP-mixed pilot site and from July 2016 to June 2018 for all remaining sites, including two additional PSP-mixed pilot sites and all non-PSP sites. A wash-out period was applied while staff were trained in the PSP and supporting infrastructure put into place. This was between September 2017 and July 2018 for the first pilot site, from June 2018 to July 2018 for the remaining two pilot sites, and from June 2018 to January 2019 for the remaining PSP and non-PSP sites.

The PSP implementation period was from July 2018 to July 2020 for the three PSP-mixed pilot sites. For the remaining sites, including PSP-CH and non-PSP sites, the PSP implementation period was from January 2019 to December 2020. A schedule is provided in Supplement 4. The wash-out period was also applied to non-PSP sites as a counterfactual condition to determine state-wide trends in healthcare utilisation independent of the program.

### Data collection

This study used available data that are routinely collected in the Queensland Hospital Admitted Patient Data Collection (QHAPDC). Data from QHAPDC were available in aggregate form as summary statistics by pre-intervention, wash-out, and intervention periods for each group, with 24 months of pre- and post-implementation data for each site. Utilisation data included: non-ICU LOS; ICU LOS; ICU admission rate; and the transfer out rate. Non-ICU LOS refers to any amount of time during admission not spent in the ICU.

### Outcomes

The primary study outcome was hospital length of stay in bed days, separated into ICU and non-ICU bed days. The value of ICU bed days selected was $5381 (SD $1423), derived from previous research^22^ and updated to 2021 values using a 3% discount rate, standard for economic evaluations in developed countries such as the USA and Australia^23,24^. The value of ward bed days could not be derived from the literature. We solicited the cost of a paediatric bed day from Queensland Health and received a mean value plus overheads of $1512 per day based on representative general wards from the hospital-based corporate information system. Due to the uncertainty around this figure, we applied the same level of uncertainty as in the ICU day valuation, which was 26.4% ($400). Interhospital transfer costs were not available, but the transfer rate was analysed for context.

### Statistical and economic analysis

Probabilistic sensitivity analysis (PSA) is a method of quantifying uncertainty in model input parameters using Monte Carlo simulation methods^25,26^. As patient-level data were not accessible to determine whether changes were due to sampling bias, simulation was applied to estimate uncertainty intervals. Summary statistics were used to create appropriate prior distributions, from which 10,000 samples were drawn to compare patient outcomes before and after implementation across PSP and non-PSP sites^25^.

To estimate statewide changes following implementation, our null hypothesis was that rates would remain unchanged. Expected results were calculated by multiplying post-implementation sample sizes by pre-implementation utilisation. Expected results were then subtracted from observed results to estimate changes associated with the PSP. To determine the counterfactual, or expected change at PSP sites in the program’s absence, difference-in-difference calculations were applied. The percentage change for non-PSP sites was subtracted from the change for PSP sites to account for general care trends across Queensland.

### Program costs

The costs of the PSP were calculated by summarizing total labour costs for clinical, administrative, and support staff to develop and deliver the program from 2017 to 2021. Staff time allocated to project implementation was tabulated for both in-kind and budgeted activities. Staff time was then multiplied by salary rates calculated pro-rata based on Queensland Health pay scales^27^ and converted to 2021 values. A detailed summary of all PSP costs is contained in Supplement 5.

## Results

Pre-implementation, there were 819 admissions (409.5/year) and an 18.2% ICU admission rate (149 admissions, or 74.5/year) in the PSP group. In the non-PSP group, there were 236 (118/year) admissions and a 13.1% ICU admission rate (31 admissions, or 15.5/year). This changed post-implementation to 1211 admissions (605.5/year) and a 17.4% ICU admission rate (211 admissions, or 105.5/year) in the PSP group. In the non-PSP group, there were 293 (146.5/year) admissions and 11.9% ICU admission rate (35 admissions, or 17.5/year). Transfers out from PSP-mixed dropped from 25.6% (140, or 70/year) to 17.0% (139, or 69.5/year) and increased in the non-PSP group from 36.0% (85, or 42.5/year) to 39.2% (115, or 57.5/year) between pre- and post-implementation.

Mean LOS dropped from 4.1 (SD 5.7) to 3.7 (SD 4.5) days for PSP-mixed, 10.2 (SD 13.9) to 8.1 (SD 9.6) days for PSP-CH, and 2.8 (SD 4.2) to 2.6 (SD 5.2) for non-PSP sites. Patient mortality in the PSP group was 1.1% (9 deaths, or 4.5/year) pre-implementation and 0.3% (2 deaths, or 1/year) post-implementation. Mortality in the non-PSP group was 0.4% (1 death, or 0.5/year) pre-implementation and 0.7% (2 deaths, or 1/year) post-implementation.

### Probabilistic sensitivity analysis

There was a high probability of reductions in LOS in both ICU and non-ICU utilisation across the PSP sites following implementation (Table 1). The most likely reductions in LOS across all PSP sites were 20.8 h in non-ICU utilisation and 9.4 h for ICU utilisation, with 99.7% and 89.8% of simulations showing a reduction, respectively. Rate of admission to ICU was likely unchanged. For non-PSP sites, the most likely changes were a reduction in non-ICU LOS of nearly 5 h and an increase in ICU LOS of over 7 h. These occurred in 74% and 66% of simulations, respectively.

**Table 1:** Results of probabilistic sensitivity analysis pre- and post-intervention.

Probabilistic sensitivity analysis		Expected value			Uncertainty interval				Pre > post
Cohort	Variable	Pre	Post	Likely change	Lower (2.5)	IQR (25.0)	IQR (75.0)	Upper (97.5)	Percent of simulations*
PSP (all)	Non-ICU LOS	5.38	4.51	− 0.86	− 1.50	− 1.08	− 0.65	− 0.33	99.7%
	ICU LOS	3.95	3.56	− 0.39	− 1.02	− 0.60	− 0.18	0.21	89.8%
	ICU admit rate	0.18	0.17	− 0.01	− 0.04	− 0.02	0.00	0.03	66.8%
Escalation (PSP-mixed to PSP-CH)	Escalation rate	0.26	0.17	− 0.09	− 0.13	− 0.10	− 0.07	− 0.04	> 99.9%
Non-PSP	Non-ICU LOS	2.90	2.71	− 0.19	− 0.79	− 0.39	0.02	0.41	73.5%
	ICU LOS	4.58	4.91	0.32	− 0.87	− 0.11	0.73	1.57	30.9%
	ICU admit rate	0.13	0.12	− 0.01	− 0.07	− 0.03	< 0.01	0.04	65.8%
Escalation (non-PSP to PSP-CH)	Escalation rate	0.36	0.39	0.03	− 0.05	< 0.01	0.06	0.11	22.3%

*Percent of simulations for which post-intervention rates were lower than pre-intervention rates for all variables. Length of stay measured in days. PSP (all)— Paediatric Sepsis Pathway. PSP-CH—Children’s hospital included in the PSP. PSP-mixed—all participating PSP sites excluding PSP-CH.

### Policy impact

There was an estimated reduction in total non-ICU days of 1013 (507.5/year, − 15.6%) at PSP sites, compared to a reduction of 55 days (27.5/year, − 6.4%) at non-PSP sites. There was also an estimated reduction in total ICU days of 111 (− 12.9%, 55.5/year), compared to an increase of 11 days (+ 7.0%, 5.5/year) at non-PSP sites. Escalations declined by 69 (− 33.5%, 34.5/year) at PSP-mixed sites, compared to an increase of 9 (+ 8.9%, 4.5/year) at non-PSP sites (Table 2).

**Table 2:** Summary of policy impacts derived from PSA.

	Group	Pre	Expected post	Observed post	Change (95% CI)	Difference-in-difference change (95% CI)
Non-ICU days	PSP	4411	6481	5467	− 1013 [− 2051, − 24]	− 596 [− 942, − 276]
	Non-PSP	682	847	793	− 55 [− 232, 120]	
ICU days	PSP	589	862	751	− 111 [− 309, 74]	− 172 [− 222, − 148]
	Non-PSP	142	160	172	11 [− 30, 55]	
Transfers	PSP-mixed	140	209	139	− 70 [− 108, − 34]	− 83 [− 97, − 83]
	Non-PSP	85	106	115	9 [− 15, 33]	

Change column refers to the difference between observed and expected outcomes. Difference-in-difference analysis subtracts non-PSP change from PSP change.

*IQR* interquartile range reflecting 25th, 75th percentile of simulations, *PSP* Paediatric Sepsis Pathway, *PSP-CH* Children’s Hospital included in the PSP, *PSP-mixed* all participating PSP sites excluding PSP-CH.

Following difference-in-difference analysis, the expected reduction in non-ICU bed days was 596 (− 9.2%, 298/year). The expected reduction in ICU bed days was 172 (− 19.9%, 86/year) and the expected reduction in transfers out was 83 (− 39.6%, 44.5/year). Confidence intervals for these figures are displayed in Table 3.

**Table 3:** Economic value of freed ICU and non-ICU bed days.

Economic valuation	Bed day value [95% CI]	Days saved* [95% CI]	Total value* [95% CI]
Non-ICU bed day	$1512 [$835, $2408]	596 [277, 942]	$901,152 [$350,945, $1,748,783]
ICU bed day	$5381 [$2950, $8503]	172 [148, 222]	$925,532 [$521,447, $1,635,807]
Total freed capacity value			$1,826,684 [$872,392, $3,384,590]

*After difference-in-difference adjustment.

### Implementation costs

The cost of program implementation over its duration was estimated at $1,729,665 (Supplement 5). The value of freed ICU and non-ICU bed days was $1,826,684 [$872,392, $3,384,590] after difference-in-difference analysis (Table 3). Thus, the most likely outcome was a cost savings of $97,019 [− $857,273, $1,654,925] for the state over 24 months ($48,510 [− $428,636, $827,463] annually), though this was subject to some uncertainty. The PSP was cost-saving in 63.4% of simulations.

## Discussion

This study used aggregate administrative hospital data before and after the implementation of a PSP across Queensland. Findings from this economic evaluation highlighted a likely reduction in utilisation rates across several key metrics. The overall policy impact of implementing the program was a substantial reduction in non-ICU and ICU utilisation, albeit subject to uncertainty. By attaching bed day values to these reductions, it was likely that the PSP was cost-neutral or slightly cost saving for the health system.

Our results show a slight reduction in length of stay for PSP-mixed sites, suggesting patients may have received necessary care sooner as early sepsis intervention is associated with reduced LOS^28^. Significantly more PSP-mixed patients were managed at their local hospital following the PSP. We hypothesize that the intervention may have given clinicians the resources and training to treat more patients locally.

### Comparisons with current literature

Current evidence for sepsis bundles shows that sepsis can be detected accurately^29^ and leads to mortality reductions in the UK^30^ and New York^28,31^ following bundle completion in adults. In children, results have been mixed^32,33^, potentially due to bundle adherence. Lower risk-adjusted in-hospital mortality following bundle application was observed in New York, but only 25% of patients received the bundle within 1 h of diagnosis^7^. Mortality rates were obtained in this study, but given the low mortality rate of paediatric sepsis patients at baseline (1.1%), we were hesitant to include the impact of mortality reductions as even small changes due to chance could be deemed significant^34^.

This is the first published study to describe systems-level changes in health care utilisation following the implementation of an ED sepsis recognition, escalation and management protocol in paediatric patients. While many studies have examined the benefits of similar programs on an individual patient basis, none have examined their results from a health system perspective using simulation to enable improved decision making. This study demonstrates that sepsis bundles may lead to modest cost savings at a systems level, though other studies show no difference^12^ or an increase^11^ in inpatient costs on a per-patient level.

Healthcare in a geographically large state such as Queensland must cater to both urban and remote regions. Centralization of services is one potential solution^35^ that has shown positive outcomes for patients^36,37^. However, centralization necessitates transfers to higher acuity hospitals, a costly process with a burden on patients and their families in a physically large state. Estimates from the UK indicate that these costs, including ongoing treatment of complications and lost productivity from delayed recognition and treatment, may significantly outweigh direct health system costs^38^. By training clinicians in remote regions to recognize and manage paediatric sepsis sooner, it appears possible to reduce the need for retrievals, enabling more local treatment without negatively affecting patient outcomes. These findings therefore may be applicable to health systems beyond the Australian context, especially in areas with a high degree of geographic remoteness, such as Russia or Canada.

### Sepsis coding

The number of paediatric sepsis diagnoses upon admission increased from 819 to 1211 in the PSP-mixed group following implementation while the non-PSP group increased from 236 to 293. Sepsis is often undercoded^39^; improved recognition upon ED presentation was a key component of the intervention, indicating increases may have been due to improved awareness of the symptoms and signs of sepsis in the ED. Changes to coding practices, a recognized phenomenon in sepsis documentation^40,41^, between pre- and post-intervention periods from the international classification of diseases may also have contributed to this change^42^. Paediatric sepsis coding has led to substantial uncertainty in the recognition and diagnosis of sepsis and severe sepsis^43^, which may have captured a lower acuity patient population. However, ICU admission rate at all sites remained relatively stable pre- and post-implementation, indicating that major changes to acuity were unlikely.

### Limitations

Constrained by time and the accessibility of patient-level data, the choice of a population-level analysis nonetheless offered the opportunity to provide robust estimates of net costs that might be expected when implementing similar programs. It is possible that the change in sepsis awareness led to lower acuity patients being diagnosed and coded as sepsis, which could reduce utilisation rates through sampling bias. However, earlier recognition may also have prevented rapid deterioration and led to a lower severity of illness through the prevention of shock. Further research on the PSP should address these limitations using patient-level data.

Accounting for additional factors such as readmission rates, improvements to morbidity and mortality, quality of life, transport costs and other measures of utility could significantly increase the value of the PSP. A study taking a societal perspective of sepsis prevention on mortality found substantial cost savings^44^.

A slight mismatch in the number of hospitals with ICU capacity in the non-PSP group may have affected validity as a counterfactual. Eight non-PSP hospitals had ICU capacity compared to 16 in the PSP group, and some quality improvement initiatives may have been targeted towards ICU-capable sites over this period. However, there may have also been initiatives targeted towards sites without ICU capability over this period, and as we examined the rate of change rather than absolute change, the risk of bias was deemed low. Unknown factors such as administrative workload may have been a possible source of confounding causing differences in patient care between ICU-capable PSP and non-PSP sites. Another potential confounder was that it was not possible to collect information on compliance with the PSP among participating sites. Re-engagement with participating sites as part of an implementation evaluation may be able to retrospectively assess compliance as well as identify any potential differences between sites implementing and not implementing the PSP. This is a topic for future research and can be informed by an audit of compliance practices and organisational factors.

While there were no other concurrent state-wide sepsis programs, quality improvement is an ongoing process that can lead to incremental improvements in care delivery over time^45^ including reductions in LOS^46^. While a greater concentration of higher capability sites in the PSP-mixed group compared to the non-PSP group may have led to improvements unrelated to the PSP, other state-wide programs may have been in place to improve the performance of lower capability sites. Future research using patient-level data and matched cases and controls could identify whether this was possible. It was also likely that there was some ‘bleeding over’ from the hospitals within the PSP to hospitals outside of it, especially between intensive and emergency practitioners who often work within multiple facilities, potentially making these findings more conservative.

This study was not able to directly calculate cost savings from a hospital perspective, instead using valuations of bed days. This may be why our results demonstrate cost savings. When re-evaluating the PSP using patient-level data, future research might incorporate National Hospital Costs Data Collection episode-level costs in a data linkage to improve estimates beyond our extrapolated average bed day values.

## Conclusion

This study observed an association between the introduction of the PSP and a reduction in healthcare utilisation. While this study shows that the PSP was likely modestly cost-saving for the health system, a patient-level statistical analysis is required to determine whether this was due to changes in case mix or a result of the PSP.

## Supplementary Information

Supplementary Information.

## Data Availability

Data is available in aggregate format (the same format accessible to the authors) in Supplement 5.

## References

[1] Singer M (2016). The third international consensus definitions for Sepsis and Septic Shock (Sepsis-3). JAMA.

[2] Rudd KE (2020). Global, regional, and national sepsis incidence and mortality, 1990–2017: Analysis for the Global Burden of Disease Study. The Lancet.

[3] Fleischmann-Struzek C (2018). The global burden of paediatric and neonatal sepsis: A systematic review. Lancet Respir. Med..

[4] Schlapbach LJ (2015). Mortality related to invasive infections, sepsis, and septic shock in critically ill children in Australia and New Zealand, 2002–13: A multicentre retrospective cohort study. Lancet. Infect. Dis..

[5] Winters BD (2010). Long-term mortality and quality of life in sepsis: A systematic review. Crit. Care Med..

[6] Simpson A (2021). Long-term functional outcomes after sepsis for adult and pediatric critical care patients—Protocol for a systematic review. Crit. Care Med..

[7] Evans IVR (2018). Association between the New York sepsis care mandate and in-hospital mortality for pediatric sepsis. JAMA.

[8] Schlapbach LJ (2019). Paediatric sepsis. Curr. Opin. Infect. Dis..

[9] Weiss SL (2020). Surviving Sepsis Campaign International Guidelines for the management of septic shock and sepsis-associated organ dysfunction in children. Pediatr. Crit. Care Med..

[10] Suarez D (2011). Cost-effectiveness of the Surviving Sepsis Campaign protocol for severe sepsis: a prospective nation-wide study in Spain. Intensive Care Med..

[11] Talmor D (2008). The costs and cost-effectiveness of an integrated sepsis treatment protocol. Crit. Care Med..

[12] Bourne DS (2020). Economic analysis of mandated protocolized sepsis care in New York Hospitals. Crit. Care Med..

[13] Jones AE, Troyer JL, Kline JA (2011). Cost-effectiveness of an emergency department-based early sepsis resuscitation protocol. Crit. Care Med..

[14] Cruz AT (2020). Updates on pediatric sepsis. J. Am. Coll. Emerg. Phys. Open.

[15] The George Institute. *Stopping Sepsis: A National Action Plan* (2017).

[16] Schlapbach, L. J., Thompson, K. & Finfer, S. R. The WHO resolution on sepsis: What action is needed in Australia? *Med. J. Aust.* **211**, 395–397e391. 10.5694/mja2.50279 (2019). 10.5694/mja2.50279 31329300

[17] Harley A (2021). Queensland Pediatric Sepsis Breakthrough Collaborative: Multi-centre observational study to evaluate the implementation of a pediatric sepsis pathway within the Emergency Department. Crit .Care Explor..

[18] Venkatesh B (2021). Impact of 1-hour and 3-hour sepsis time bundles on patient outcomes and antimicrobial use: A before and after cohort study. Lancet Regional Health West. Pac..

[19] Husereau D (2013). Consolidated Health Economic Evaluation Reporting Standards (CHEERS) statement. Value Health.

[20] Health, Q. *About the CSCF*. https://www.health.qld.gov.au/clinical-practice/guidelines-procedures/service-delivery/cscf/about (2016).

[21] Queensland Government Statistician’s Office. *Population Growth Highlights and Trends*, Queensland, 2021 edition. (2021).

[22] Hicks P (2019). The financial cost of intensive care in Australia: a multicentre registry study. Med. J. Aust..

[23] Gold MR (1996). Cost-Effectiveness in Health and Medicine.

[24] Haacker M, Hallett TB, Atun R (2020). On discount rates for economic evaluations in global health. Health Policy Plan..

[25] Briggs A, Sculpher M, Claxton K (2006). Decision Modelling for Health Economic Evaluation.

[26] Briggs AH (2000). Handling uncertainty in cost-effectiveness models. Pharmacoeconomics.

[27] Government, Q. *Queensland Health Wage Rates*. https://www.health.qld.gov.au/hrpolicies/wage-rates (2021).

[28] Leisman D (2016). Association of fluid resuscitation initiation within 30 minutes of severe sepsis and septic shock recognition with reduced mortality and length of stay. Ann. Emerg. Med..

[29] Balamuth, F. *et al.* Improving recognition of pediatric severe sepsis in the Emergency Department: Contributions of a vital sign-based electronic alert and bedside clinician identification. *Ann. Emerg. Med.* **70**, 759–768e752. 10.1016/j.annemergmed.2017.03.019 (2017). 10.1016/j.annemergmed.2017.03.019 PMC5698118 28583403

[30] Daniels R, Nutbeam T, McNamara G, Galvin C (2011). The sepsis six and the severe sepsis resuscitation bundle: A prospective observational cohort study. Emerg Med J.

[31] Seymour CW (2017). Time to treatment and mortality during mandated emergency care for sepsis. N. Engl. J. Med..

[32] Lane RD, Funai T, Reeder R, Larsen GY (2016). High reliability pediatric septic shock quality improvement initiative and decreasing mortality. Pediatrics.

[33] Paul R (2018). A quality improvement collaborative for pediatric sepsis: lessons learned. Pediatr. Qual. Saf..

[34] King G, Zeng L (2001). Logistic regression in rare events data. Polit. Anal..

[35] Ostermann M, Vincent JL (2019). How much centralization of critical care services in the era of telemedicine?. Crit Care.

[36] Pearson G (1997). Should paediatric intensive care be centralised? Trent versus Victoria. Lancet.

[37] Roussak P (2014). Centralisation of paediatric intensive care and a 24-hour retrieval service. Br J Nurs.

[38] Hex, N., Retzler, J., Bartlett, C., & Arber, M. *The Cost of Sepsis Care in the UK* (2017).

[39] Jolley RJ (2015). Validation and optimisation of an ICD-10-coded case definition for sepsis using administrative health data. BMJ Open.

[40] Rhee C (2017). Incidence and trends of sepsis in US hospitals using clinical vs claims data, 2009–2014. JAMA.

[41] Rudd KE, Delaney A, Finfer S (2017). Counting sepsis, an imprecise but improving science. JAMA.

[42] Jordan Kempker, A., Rudd, K. E., Wang, H. E. & Martin, G. S. Sepsis epidemiology across the international classification of diseases, 9th edition, to international classification of diseases, 10th Edition, Chasm-A direct application of the institute for health metrics and evaluation case definition to hospital discharge data. *Crit. Care Med.* **48**, 1881–1884. 10.1097/ccm.0000000000004577 (2020). 10.1097/CCM.0000000000004577 PMC11904614 33009097

[43] Schlapbach LJ, Kissoon N (2018). Defining pediatric sepsis. JAMA Pediatr..

[44] Khowaja AR (2021). The return on investment of a province-wide quality improvement initiative for reducing in-hospital sepsis rates and mortality in British Columbia, Canada. Crit. Care Med..

[45] Escobar GJ, Plimier C, Greene JD, Liu V, Kipnis P (2019). Multiyear rehospitalization rates and hospital outcomes in an integrated health care system. JAMA Netw. Open.

[46] Australian Institute of Health and Welfare. *Hospital Care* (AIHW, 2020).

