# Supplementary material for: Patient and economic impact of implementing a paediatric sepsis pathway in emergency departments in Queensland, Australia
Source: Sci Rep. 2022 Jun 16;12:10113. doi: 10.1038/s41598-022-14226-6 (PMC9203710; doi:10.1038/s41598-022-14226-6)
Supplement: Supplementary file 1 — Supplementary Information. [file 41598_2022_14226_MOESM1_ESM.pdf]

Supplement 1

Queensland Sepsis Breakthrough Collaborative authorship:

Queensland Children's Hospital
A/Prof Luregn Schlapbach, PICU Staff Specialist, past lead QPSP
Ms Amanda Harley, Pediatric Sepsis Clinical Nurse Consultant
Dr Adam Irwin, Infectious Disease Staff Specialist, present medical co-lead QPSP
Ms Nicolette Graham, Senior Antimicrobial Stewardship Pharmacist
Dr Fiona Thomson, Emergency Department Staff Specialist
Mr Kieran Owen, Sepsis Clinical Nurse
Ms Kirsten Garrish, Emergency Department Clinical Nurse
Ms Emma Sampson, Emergency Department Clinical Nurse
A/Prof Debbie Long, PICU Nurse Researcher

Gold Coast University and Robina Hospital
Dr Shane George, Emergency Department Staff Specialist
Prof Keith Grimwood, Infectious Diseases Staff Specialist
Dr Christa Bell, Emergency Department Staff Specialist
Ms Bethany Semple, Clinical Nurse
Ms Claire Adams, Clinical Nurse
Ms Josea Brown, Clinical Nurse
Ms Louise Maloney, Clinical Nurse

Sunshine Coast Universtiy Hospital/Nambour Hospital
A/Prof Paula Lister, Director of Pediatric Intensive Care, present medical co-lead QPSP.
Dr Scott Schofield Emergency Department Staff Specialist
Dr Clare Thomas, Pediatric Staff Specialist
Mr Liam Dejong, Clinical Nurse
Ms Esther Bently, Clinical Nurse

Cairns Hospital
Dr Lambros Halkidis, Emergency Department Staff Specialist
Dr Cheryl Bird, Emergency Department Staff Specialist
Mr Matthew Smith, Clinical Nurse Consultant
Ms Pia Alexander, Clinical Nurse
Laura Davidson-West, Registered Nurse

Rockhampton Hospital
Dr Titiosibina Ebenezer Adegbija, Emergency Department Staff Specialist
Ms Alice Brandt, Emergency Department Nurse Educator
Ms Bree Walker, Director Patient Safety
Ms Andrea McLucas, ICU Nurse Educator

Bundaberg Hospital
Dr Adam Philip Michael, Emergency Department Staff Specialist
Ms Samantha Hoole, Emergency Department Nurse Educator
Ms Candice Bauer, Registered Nurse

Redland Hospital
Dr John Sutherland, Director of ED
Dr Douglas Gordon Thomas, Director of Pediatrics
Dr David Van der Walt, Emergency Department Staff Specialist
Ms Jessica Hulme, Clinical Facilitator
Ms Kerrie Burke, Clinical Nurse Consultant Patient Safety

Redcliffe Hospital

Ms Helena Cooney, Sepsis Clinical Nurse Consultant
Dr Doug Morel, Emergency Department Staff Specialist
Ms Louise O’Riordan, Director Safety and Quality

Ipswich Hospital
Dr Samantha Fairless, Emergency Department Staff Specialist
Ms Megan Bool, Clinical Nurse Consultant

Logan Hospital
Dr Nandini Choudary, Emergency Department Staff Specialist
Dr Shalini Arora, Emergency Department Staff Specialist
Dr Ben Lawton, Emergency Department Staff Specialist
Ms Jo Farrell, Clinical Nurse Consultant

Hervey Bay Hospital
Dr Penelope Prasad, Emergency Department Staff Specialist
Dr Rudesh Prasad, Emergency Department Staff Specialist
Ms Laura O’Connor, Registered Nurse
Mr Timothy Butters, Registered Nurse

The Prince Charles Hospital
Mr Peter Kennedy, Clinical Nurse
Dr Hanh Pham, Emergency Department Staff Specialist

Caboolture Hospital
Dr Maya Aoude, Emergency Department
Ms Sara Blundell, Emergency Department Clinical Nurse
Ms Natasha Willmett, Emergency Department Clinical Nurse

Mackay Hospital
Ms Louise McGrath, Nurse Unit Manager
Ms Karen Smith, Clinical Nurse Consultant

Clinical Excellence Queensland
Ms Kate Weller, Manager, Project Manager
Ms Trina Maturanec, Project Manager
Michael Rice (Chair)
Balasubramanian Venkatesh (Chair)
Paul Lane, Medical Co-lead
Robert Seaton, Data analyst
Donna Mason, Principal Project Officer
Naitik Mehta, Principal Project Officer
Vikrant Kalke, Principal Project Officer
Damien Jones, Consumer Representative
Mathew Ames, Consumer Representative
Mary Steele, Consumer Representative
Amy Wilkinson, Consumer Representative

The University of Queensland
A/Prof Kristen Gibbons, Senior Epidemiologist, Child Health Research Centre, Faculty of Medicine
Ms Patricia Gilholm, Data Scientist, Child Health Research Centre, Faculty of Medicine
Mr Endrias Ergetu, Data Analyst, Child Health Research Centre, Faculty of Medicine
Ms Rachel Treadwell, Student Nurse/Midwifery
Ms Tahlia Van Raders, Student Nurse/Midwifery
Ms Jessicah Minogue, Master of Philosophy Student

Supplement 2

Table S2: CHEERS checklist 2022

Topic	No.	Item	Location where item is reported
**Title**			
	1	Identify the study as an economic evaluation and specify the interventions being compared.	Title
**Abstract**			
	2	Provide a structured summary that highlights context, key methods, results, and alternative analyses.	Abstract
**Introduction**			
**Background and objectives**	3	Give the context for the study, the study question, and its practical relevance for decision making in policy or practice.	Introduction (throughout)
**Methods**			
**Health economic analysis plan**	4	Indicate whether a health economic analysis plan was developed and where available.	N/A
**Study population**	5	Describe characteristics of the study population (such as age range, demographics, socioeconomic, or clinical characteristics).	Methods (Setting and population)
**Setting and location**	6	Provide relevant contextual information that may influence findings.	Methods (Setting and population)
**Comparators**	7	Describe the interventions or strategies being compared and why chosen.	Methods (Setting and population, Statistical and economic analysis)
**Perspective**	8	State the perspective(s) adopted by the study and why chosen.	Methods (Statistical and economic analysis)
**Time horizon**	9	State the time horizon for the study and why appropriate.	Methods (Program costs)
**Discount rate**	10	Report the discount rate(s) and reason chosen.	Methods (Outcomes)
**Selection of outcomes**	11	Describe what outcomes were used as the measure(s) of benefit(s) and harm(s).	Methods (Outcomes)
**Measurement of outcomes**	12	Describe how outcomes used to capture benefit(s) and harm(s) were measured.	Methods (Data collection)

Topic	No.	Item	Location where item is reported
**Valuation of outcomes**	13	Describe the population and methods used to measure and value outcomes.	Methods (Statistical and economic analysis)
**Measurement and valuation of resources and costs**	14	Describe how costs were valued.	Methods (Outcomes, Program costs)
**Currency, price date, and conversion**	15	Report the dates of the estimated resource quantities and unit costs, plus the currency and year of conversion.	Methods (Outcomes)
**Rationale and description of model**	16	If modelling is used, describe in detail and why used. Report if the model is publicly available and where it can be accessed.	Methods (Statistical and economic analysis)
**Analytics and assumptions**	17	Describe any methods for analysing or statistically transforming data, any extrapolation methods, and approaches for validating any model used.	Methods (Statistical and economic analysis)
**Characterising heterogeneity**	18	Describe any methods used for estimating how the results of the study vary for subgroups.	Methods (Statistical and economic analysis)
**Characterising distributional effects**	19	Describe how impacts are distributed across different individuals or adjustments made to reflect priority populations.	Supplement 2
**Characterising uncertainty**	20	Describe methods to characterise any sources of uncertainty in the analysis.	Methods (Statistical and economic analysis)
**Approach to engagement with patients and others affected by the study**	21	Describe any approaches to engage patients or service recipients, the general public, communities, or stakeholders (such as clinicians or payers) in the design of the study.	N/A
**Results**			
**Study parameters**	22	Report all analytic inputs (such as values, ranges, references) including uncertainty or distributional assumptions.	Results, Supplement 2
**Summary of main results**	23	Report the mean values for the main categories of costs and outcomes of interest and summarise them in the most appropriate overall measure.	Results
**Effect of uncertainty**	24	Describe how uncertainty about analytic judgments, inputs, or projections affect findings. Report the effect of choice of discount rate and time horizon, if applicable.	Results (Probabilistic sensitivity analysis)

Topic	No.	Item	Location where item is reported
**Effect of engagement with patients and others affected by the study**	25	Report on any difference patient/service recipient, general public, community, or stakeholder involvement made to the approach or findings of the study	Discussion (Comparisons with current literature)
**Discussion**			
**Study findings, limitations, generalisability, and current knowledge**	26	Report key findings, limitations, ethical or equity considerations not captured, and how these could affect patients, policy, or practice.	Discussion (throughout)
**Other relevant information**			
**Source of funding**	27	Describe how the study was funded and any role of the funder in the identification, design, conduct, and reporting of the analysis	Acknowledgements
**Conflicts of interest**	28	Report authors conflicts of interest according to journal or International Committee of Medical Journal Editors requirements.	Competing interests

From: Husereau D, Drummond M, Augustovski F, et al. Consolidated Health Economic Evaluation Reporting Standards 2022 (CHEERS 2022) Explanation and Elaboration: A Report of the ISPOR CHEERS II Good Practices Task Force. Value Health 2022;25. doi:10.1016/j.jval.2021.10.008

Supplement 3

Definitions used for data collection
CSCF ED 3+
Queensland Health level 3 emergency care centers and higher. All hospitals included in the analysis (16 PSP sites and 32 non-PSP sites) included in this group:

- "…Provides on-site, 24- hour access with designated emergency registered nurse and triage of all presentations.
- Capable of providing initial treatment and care for all presentations, and advanced resuscitation and stabilisation, including short-term assisted ventilation prior to transfer to higher-level service.
- Ability to assist in care of minor trauma and enable rapid transfer of major trauma."

Population
Every case at public Queensland Health facilities (public acute hospitals, public psychiatric hospitals, and primary health clinics, excluding residential care and mental health facilities)

- Over the date ranges
- Aged 0-17 years
- With any of 45 sepsis international classification of disease 10 (ICD-10) codes as principal or secondary diagnosis recorded as 'present on admission' after presentation through ED at the same facility (listed as community onset sepsis)
    - Cases with only ward-acquired sepsis or inbound transfers that circumvented the ED were excluded

No sampling was used for this population; dataset included all patients defined above as the population within the specified date ranges.

Sepsis coding practices in ICD-10

```
** Sepsis ICD codes;
%let Sepsis_ICD_list =
'A021', /*Salmonella sepsis*/
'A207', /*Septicaemic plague*/
'A227', /*Anthrax sepsis*/
'A241', /*Acute and fulminating melioidosis*/
'A267', /*Erysipelothrix sepsis*/
'A327', /*Listerial sepsis*/
'A40', /*Streptococcal sepsis*/
'A400', /*Sepsis due to streptococcus, group A*/
'A401', /*Sepsis due to streptococcus, group B*/
'A402', /*Sepsis due to streptococcus, group D*/
'A403', /*Sepsis due to Streptococcus pneumoniae*/
'A408', /*Other streptococcal sepsis*/
'A409', /*Streptococcal sepsis, unspecified*/
'A41', /*Other sepsis*/
'A410', /*Sepsis due to Staphylococcus aureus*/
'A411', /*Sepsis due to other specified staphylococcus*/
'A412', /*Sepsis due to unspecified staphylococcus*/
'A413', /*Sepsis due to Haemophilus influenzae*/
'A414', /*Sepsis due to anaerobes*/
'A415', /*Sepsis due to other Gram-negative organisms*/
'A4150', /*Sepsis due to unspecified Gram-negative organisms*/
'A4151', /*Sepsis due to Escherichia coli [E. Coli]*/
'A4152', /*Sepsis due to Pseudomonas*/
'A4158', /*Sepsis due to other Gram-negative organisms*/
'A418', /*Other specified sepsis*/
'A419', /*Sepsis, unspecified*/
'A427', /*Actinomycotic sepsis*/
'A483', /*Toxic Shock syndrome*/
'B007', /*Disseminated herpesviral disease, Herpesviral sepsis*/
'B377', /*Candidal sepsis*/
'O85', /*Puerperal sepsis */
'O883', /*Obstetric pyaemic and septic embolism*/
'P36', /*Bacterial sepsis of newborn*/
'P360', /*Sepsis of newborn due to streptococcus, group B*/
'P361', /*Sepsis of newborn due to other and unspecified streptococci*/
'P362', /*Sepsis of newborn due to Staphylococcus aureus*/
'P363', /*Sepsis of newborn due to other and unspecified staphylococci*/
'P364', /*Sepsis of newborn due to Escherichia coli*/
'P365', /*Sepsis of newborn due to anaerobes*/
'P368', /*Other bacterial sepsis of newborn*/
'P369', /*Bacterial sepsis of newborn, unspecified*/
2
'R572', /*Septic shock*/
'R650', /*Systemic inflammatory response syndrome [SIRS] of infectious
origin without acute organ failure*/
'R651', /*Severe sepsis*/
'T8142' /*Sepsis following a procedure*/
```

Supplement 4

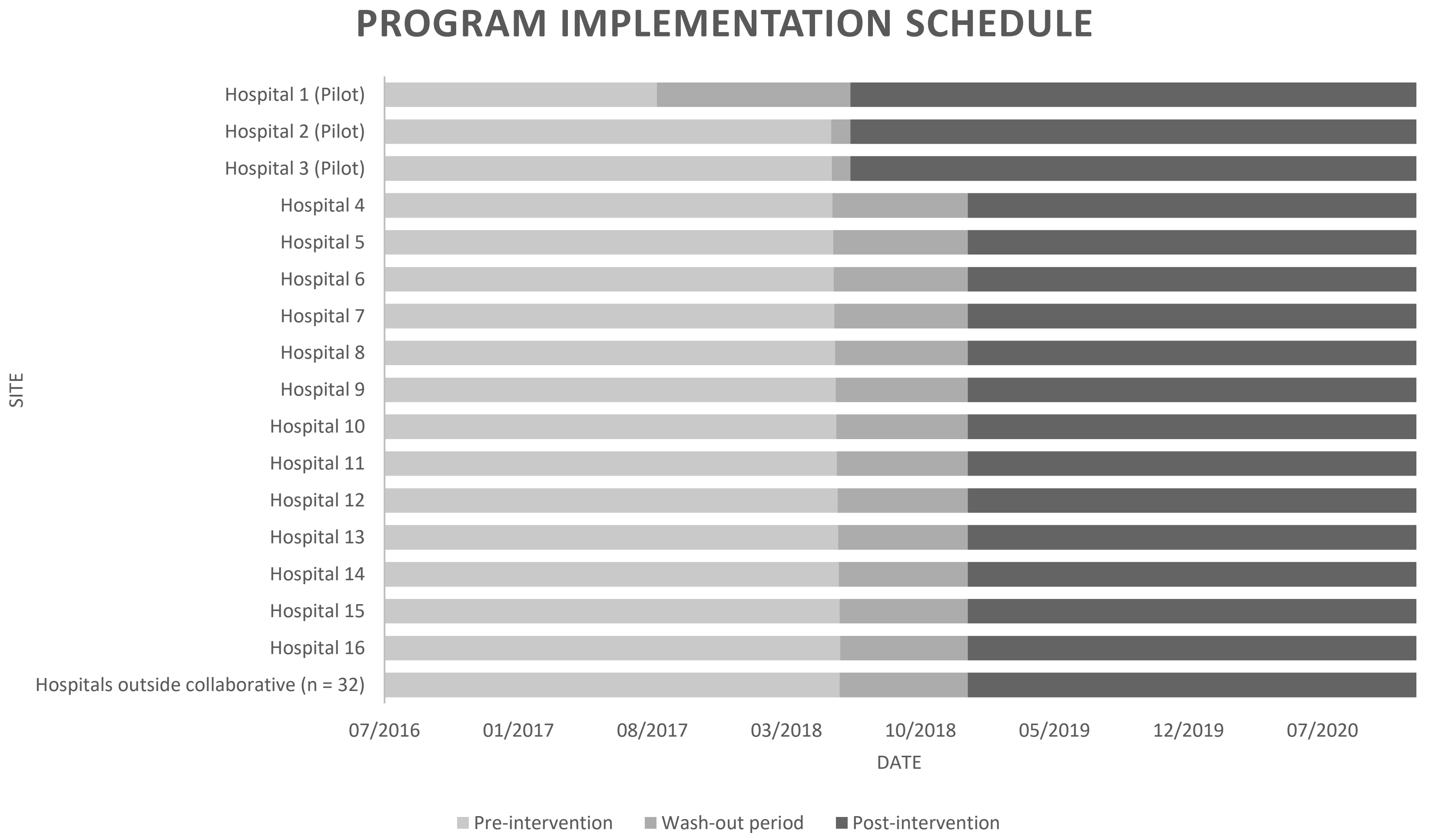

Figure S4: Implementation of the PSP by site. The start of the pre-intervention date for hospital 1 was September 2015.

Supplement 5

Implementation costs

Labor costs were calculated by matching staffing hours to designated activities of the sepsis pathway. Staff were matched to their pro-rata salary band, with hospital costs split by site depending on the ratio of pediatric and adult patients. Costs were inflated to 2021 values using a 3% discount rate. Table S1 demonstrates the cost of each labor component of the intervention.

Table S5.1: Annual costs ($AUD) for labor required to implement the sepsis pathway, adjusted to 2021 values using 3% discount rates

**Activity**	**Cost**			
	**2017-18**	**2018-19**	**2019-20**	**Total**
Data analysis, communications		$30,596	$15,914	$46,510
Pathway preparation and development	$225,102			$225,102
Social worker support	$30,389	$35,514	$18,035	$83,938
Pilot program and ongoing education package	$41,644	$41,524	$106,090	$189,257
Hospital implementation costs		$329,850	$94,841	$424,691
Clinical nurse consultant	$94,543	$190,134	$142,161	$426,838
Administration support		$77,584		$77,584
Travel and education		$72,120	$53,045	$125,165
Medical lead		$90,696		$90,696
Pharmacy lead		$39,885		$39,885
**Total**	**$391,677**	**$907,902**	**$430,085**	**$1,729,665**

Model selection

Simulation techniques examine the likelihood that the same results may be observed in other populations, such as future pediatric sepsis populations in Queensland or abroad, and to conceptualize error terms in a way that enables health policy decision making. Probabilities were transformed into Beta distributions using events and non-events. Length of stay (LOS) was transformed into Gamma distributions using means and standard error. As summary statistics were the data items available for this study, the method of moments was used as it provides a consistent estimation method for model parameters. In contrast to methods comparing sample means, this method examines the full distribution of simulated results to determine the certainty of a change in outcomes, with certainty illustrated by directly comparing a sample from each distribution for each simulation. This methodology may be referred to as a Monte Carlo bootstrap, as it applies a similar technique to bootstrapping but using sample values from a proposed distribution generated by the data (Monte Carlo), rather than sampling the data itself, in order to improve generalizability.

Model parameters

Table S5.2: Aggregate data used for analysis and relevant transformations

		Pre					Post				
		Mean	SE	Distribution	Param1	Param2	Mean	SE	Distribution	Param1	Param2
PSP-mixed	ICU admit rate	0.1463	0.0151	Beta	80.0000	467.0000	0.1285	0.0117	Beta	105.0000	712.0000
	ICU LOS	2.3661	0.1859	Gamma	161.9441	0.0146	2.9880	0.1761	Gamma	287.9995	0.0104
	non-ICU LOS	3.7083	0.2116	Gamma	307.0114	0.0121	3.3164	0.1269	Gamma	683.2020	0.0049
	Transfer rate (QCH)	0.2559	0.0187	Beta	140.0000	407.0000	0.1701	0.0132	Beta	139.0000	678.0000
	Pre-transfer LOS	2.0657	0.1682	Gamma	150.8057	0.0137	1.8143	0.1132	Gamma	256.8102	0.0071
	Mortality rate	0.0091	0.0041	Beta	5.0000	542.0000	0.0025	0.0017	Beta	2.0000	810.0000
PSP-CH	ICU admit rate	0.2537	0.0264	Beta	69.0000	203.0000	0.2690	0.0224	Beta	106.0000	288.0000
	ICU LOS	5.7887	0.6128	Gamma	89.2410	0.0649	4.1233	0.2612	Gamma	249.2719	0.0165
	non-ICU LOS	8.7705	0.6609	Gamma	176.0846	0.0498	7.0031	0.4278	Gamma	267.9850	0.0261
	Transfer rate (home)	0.0404	0.0120	Beta	11.0000	261.0000	0.0431	0.0102	Beta	17.0000	377.0000
	Pre-transfer LOS	3.6758	0.3372	Gamma	118.8276	0.0309	6.7265	0.7432	Gamma	81.9079	0.0821
	Mortality rate	0.0147	0.0073	Beta	4.0000	268.0000	0.0051	0.0036	Beta	2.0000	392.0000
PSP (all)	ICU admit rate	0.1819	0.0135	Beta	149.0000	670.0000	0.1742	0.0109	Beta	211.0000	1000.0000
	ICU LOS	3.9511	0.2706	Gamma	213.2599	0.0185	3.5583	0.1474	Gamma	582.9473	0.0061
	non-ICU LOS	5.3895	0.2738	Gamma	387.3877	0.0139	4.5159	0.1707	Gamma	700.1007	0.0065
	Transfer rate (QCH)	0.1844	0.0136	Beta	151.0000	668.0000	0.1288	0.0096	Beta	156.0000	1055.0000
	Pre-transfer LOS	2.1830	0.1423	Gamma	235.3882	0.0093	2.3496	0.1679	Gamma	195.7794	0.0120
	Mortality rate	0.0110	0.0036	Beta	9.0000	810.0000	0.0033	0.0017	Beta	4.0000	1207.0000

Non-PSP	ICU admit rate	0.1314	0.0220	Beta	31.0000	205.0000	0.1195	0.0190	Beta	35.0000	258.0000
	ICU LOS	4.5803	0.3602	Gamma	161.6780	0.0283	4.8915	0.5034	Gamma	94.4283	0.0518
	non-ICU LOS	2.8934	0.2099	Gamma	190.0780	0.0152	2.7044	0.2231	Gamma	146.9406	0.0184
	Transfer rate (QCH)	0.3602	0.0313	Beta	85.0000	151.0000	0.3925	0.0286	Beta	115.0000	178.0000
	Pre-transfer LOS	0.8545	0.1111	Gamma	59.1320	0.0145	0.8609	0.2599	Gamma	10.9715	0.0785
	Mortality rate	0.0042	0.0042	Beta	1.0000	235.0000	0.0069	0.0049	Beta	2.0000	287.0000

Subgroup analysis

Table S5.3: All results from probabilistic sensitivity analysis, split by subgroup

**Probabilistic Sensitivity Analysis**		**Expected value**			**Pre > Post**		
**Cohort**	**Variable**	**Pre**	**Post**	**Likely change**	**Lower (2.5)**	**Upper (97.5)**	**Certainty of reduction**
PSP (all)	ICU admit rate	0.1820	0.1743	-0.0077	-0.0419	0.0260	66.80%
	ICU LOS	3.9507	3.5600	-0.3908	-1.0177	0.2084	89.80%
	non-ICU LOS	5.3826	4.5158	-0.8668	-1.5023	-0.3333	99.70%
	Mortality rate	0.0110	0.0033	-0.0077	-0.0163	-0.0004	98.00%
PSP-mixed	ICU admit rate	0.1460	0.1285	-0.0175	-0.0557	0.0192	82.00%
	ICU LOS	2.3650	2.9886	0.6236	0.1141	1.1194	0.90%
	non-ICU LOS	3.7044	3.3185	-0.3859	-0.8788	0.0893	94.10%
	Escalation rate	0.2560	0.1702	-0.0859	-0.1316	-0.0416	>99.99%
	Pre-escalation LOS	2.0635	1.8138	-0.2498	-0.6518	0.1416	89.30%
	Mortality rate	0.0092	0.0025	-0.0067	-0.0165	0.0010	95.80%
PSP-CH	ICU admit rate	0.2539	0.2694	0.0155	-0.052	0.0805	32.20%
	ICU LOS	5.7898	4.1218	-1.6680	-3.0262	-0.4125	99.70%
	non-ICU LOS	8.7670	6.9956	-1.7714	-3.3823	-0.2464	99.00%
	De-escalation rate	0.0406	0.0432	0.0027	-0.0289	0.0333	42.30%
	Pre- de-escalation LOS	3.6714	6.7305	3.0591	1.5213	4.7102	<0.01%
	Mortality rate	0.0147	0.0051	-0.0096	-0.0279	0.0040	91.20%
Non-PSP	ICU admit rate	0.1314	0.1195	-0.0119	-0.0695	0.0439	65.80%
	ICU LOS	4.5842	4.9062	0.3220	-0.8699	1.5725	30.90%
	non-ICU LOS	2.8918	2.7056	-0.1862	-0.7909	0.4091	73.50%
	Escalation rate	0.3603	0.3923	0.0321	-0.0496	0.1138	22.30%
	Pre-escalation LOS	0.8538	0.8598	0.0061	-0.4871	0.6220	52.10%
	Mortality rate	0.0043	0.0069	0.0026	-0.0107	0.0163	31.30%
